# Wolverine behavior varies spatially with anthropogenic footprint: implications for conservation and inferences about declines

**DOI:** 10.1002/ece3.1921

**Published:** 2016-02-09

**Authors:** Frances E. C. Stewart, Nicole A. Heim, Anthony P. Clevenger, John Paczkowski, John P. Volpe, Jason T. Fisher

**Affiliations:** ^1^ School of Environmental StudiesUniversity of Victoria3800 Finnerty Rd.VictoriaBCCanadaV8W 2Y2; ^2^ Western Transportation InstituteMontana State UniversityPO Box 174250BozemanMontana59717; ^3^ Alberta Environment and ParksParks DivisionKananaskis Region, Suite 201800 Railway AvenueCanmoreABCanadaT1W 1P1; ^4^ Ecosystem Management UnitAlberta Innovates‐Technology Futures3‐4476 Markham St.VictoriaBCCanadaV8Z 7X8

**Keywords:** Camera trapping, *Gulo gulo*, human footprint, landscape of fear, *Mustelidae*, neophobia

## Abstract

Understanding a species’ behavioral response to rapid environmental change is an ongoing challenge in modern conservation. Anthropogenic landscape modification, or “human footprint,” is well documented as a central cause of large mammal decline and range contractions where the proximal mechanisms of decline are often contentious. Direct mortality is an obvious cause; alternatively, human‐modified landscapes perceived as unsuitable by some species may contribute to shifts in space use through preferential habitat selection. A useful approach to tease these effects apart is to determine whether behaviors potentially associated with risk vary with human footprint. We hypothesized wolverine (*Gulo gulo*) behaviors vary with different degrees of human footprint. We quantified metrics of behavior, which we assumed to indicate risk perception, from photographic images from a large existing camera‐trapping dataset collected to understand wolverine distribution in the Rocky Mountains of Alberta, Canada. We systematically deployed 164 camera sites across three study areas covering approximately 24,000 km^2^, sampled monthly between December and April (2007–2013). Wolverine behavior varied markedly across the study areas. Variation in behavior decreased with increasing human footprint. Increasing human footprint may constrain potential variation in behavior, through either restricting behavioral plasticity or individual variation in areas of high human impact. We hypothesize that behavioral constraints may indicate an increase in perceived risk in human‐modified landscapes. Although survival is obviously a key contributor to species population decline and range loss, behavior may also make a significant contribution.

## Introduction

Wildlife populations around the globe are experiencing declines and range contractions (Butchart et al. 2010) where habitat loss and fragmentation are major causes (Fahrig 1997, 2001, 2003). Human exploitation of landscapes is a consequence of increasing human populations and resource development (Woodroffe et al. 2005). Although expanding landscape modification, or “human footprint,” is well documented as a major cause of mammalian range contraction and declines in biodiversity (Vitousek et al. 1997; Laliberte and Ripple 2004), there is little research on the effects of footprint on other important aspects of species persistence such as life‐history traits, population dynamics, and behavior (Frid and Dill 2002; Béchet et al. 2004; Ciuti et al. 2012a). Human landscape modification is expected to shift species’ selection to habitats possessing the highest realized suitability (Rosenzweig 1981; Petit and Petit 1996; Abrams 2000), and this can occur by inducing mortality, or by decreasing its suitability relative to other patches available for choosing. Given rapidly expanding global human footprint (Vitousek et al. 1997), and associated ongoing mammalian population declines (Woodroffe 2000; Laliberte and Ripple 2004), it is important to assess the effects of human footprint on species of critical conservation concern.

Testing these premises for some taxa, for example, large wide‐ranging carnivores, has proved logistically difficult (but see Woodroffe 2000). However, we can make inferences by investigating shifts in behavior over large spatial scales – shifts assumed to be associated with underlying changes in human footprint. We examined spatial patterns in behavior of wolverines (*Gulo gulo*), a species of Special Concern in Canada and recently petitioned for listing under the US Endangered Species Act. Based on an analysis of camera‐trap data collected to understand wolverine distribution, we formed the post hoc hypothesis that wolverines exhibit changes in behavior across areas with varying degrees of human footprint. We suggest spatial variation in behavior may indicate variation in perceived suitability, which could be contributing to decreased distribution.

Our assumptions about wolverine behavior derive from the landscape of fear (LOF) hypothesis, applied to human‐modified landscapes. In LOF, habitat selected by a species consists of high‐ and low‐risk habitat patches, characterized by the occupancy and perceived lethality of a predator (or other source of mortality) within those patches (Caraco et al. 1980; Baker and Brown 2010; Laundre et al. 2014). Predators can cause direct mortality, but predators can also affect prey distribution through sublethal effects, which may surpass mortality in impact by manipulating prey morphology, physiology, behavior, or habitat selection (Sih et al. 1985; Sheriff et al. 2009; Ford et al. 2014). The LOF hypothesis predicts that patches with greater perceived risk will be less likely occupied, and if occupied, it will invoke increased behaviors indicative of perceived risk. A well‐known example is the trophic cascade resulting from the risk‐sensitive foraging behavior of elk following wolf re‐introduction in Yellowstone National Park (Ripple and Beschta 2006). In human‐modified landscapes, perceived risk can be manipulated by human presence and landscape modifications (Darimont et al. 2009; Ciuti et al. 2012a,b).

The perceived risk induced by human footprint can be assessed by quantifying animal behaviors (Lima and Dill 1990; Lima and Zollner 1996; Ciuti et al. 2012a) across a range of human footprints. Behavior is affected by several factors including habitat characteristics (Hollén et al. 2011), sex and density (Childress and Lung 2003), predator presence (Morrison 2011), and breeding season (Wolff and Van Horn 2003), but importantly it can correlate strongly to human presence (Wang et al. 2011). The majority of past research has been conducted on prey species, but risk concepts outlined under the LOF hypothesis could apply to carnivore species assuming they perceive cues induced by landscape modification as tradeoffs between mortality risk, competition with other carnivores, and foraging (Murphy et al. 1995). We tested this premise using wolverines distributed across landscapes of widely varying human footprints in the Rocky Mountains of Alberta, Canada.

Wolverines have experienced considerable reductions in much of their North American range over the last two centuries (Laliberte and Ripple 2004). In western Canada, wolverines are designated “Special Concern,” and remain listed as “Data Deficient” in Alberta (COSEWIC Annual Report 2014). Despite small and relatively isolated populations, wolverines in the USA remain unlisted under the Endangered Species Act (U.S. Fish and Wildlife Service 2014) in part due to debate over the cause of population decline. Several competing hypotheses exist. Wolverines are more likely to occur in areas of low human footprint (Krebs et al. 2007; Heinemeyer 2013; Fisher et al. 2013), implicating landscape development as a source of landscape change. Alternatively, wolverines are more likely to occur in areas of persistent spring snow (Copeland et al. 2010; McKelvey et al. 2011; Inman et al. 2012; Clevenger and Baruetto 2014), ostensibly because they require snow dens to raise young (Magoun and Copeland 1998). In this event, climate change may have led to population declines (Brodie and Post 2009) and may continue to do so. Hunting and trapping (Krebs et al. 2004; Lofroth and Ott 2007) may play a role, and emerging evidence suggests other carnivore competitors may also have an effect (Mattisson et al. 2011; Heim 2015). However, the proximate cause(s) of wolverines’ range contraction remain in question. Most studies have examined wolverine habitat selection or distribution, and related this to landscape characteristics such as anthropogenic disturbance as a basis for inference. We try a different approach and hypothesize that wolverine behaviors spatially vary with the degree of anthropogenic landscape footprint. We predicted that wolverines in human‐modified landscapes would be more likely to express behaviors assumed to be associated with perceived risk, than those in protected landscapes with much less landscape modification.

## Materials and Methods

### Study area

**Figure 1:**
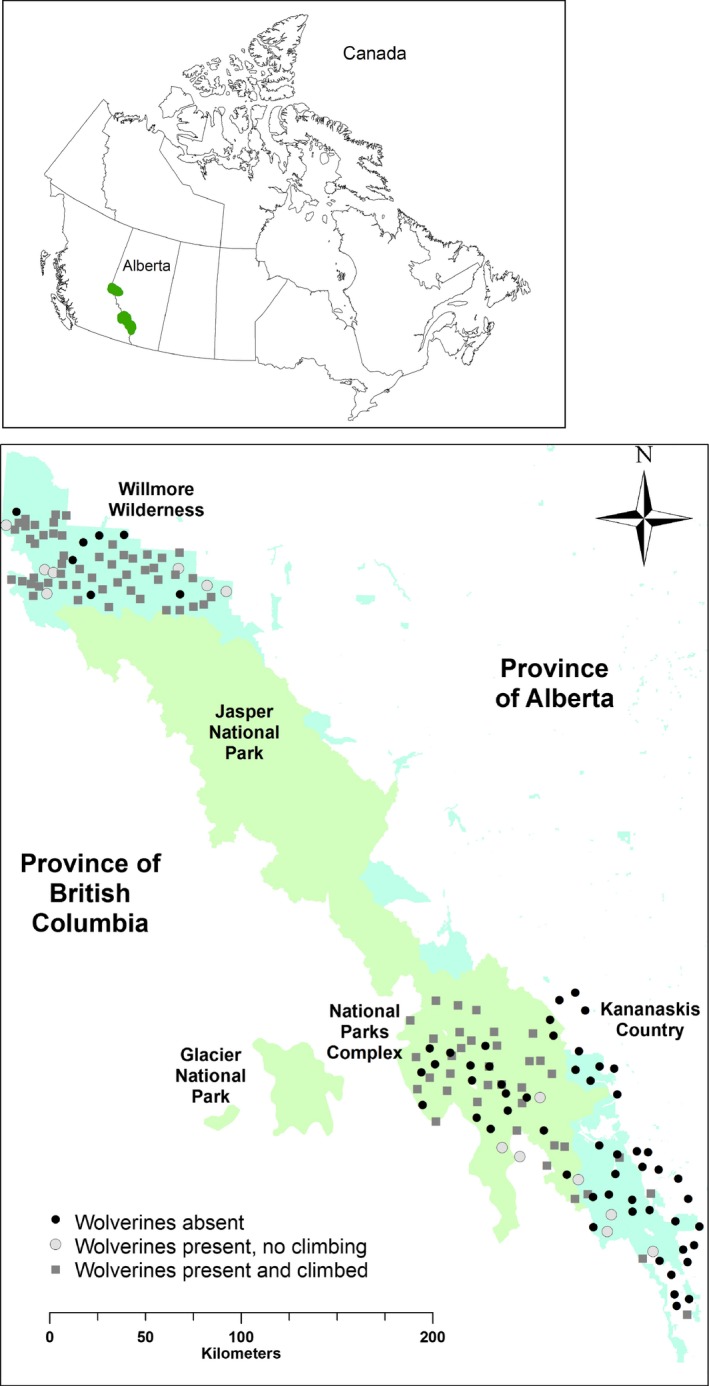
Wolverine occurrence and climbing of a baited tree were sampled across three large areas of varying human footprint and protection status. Provincial protected areas, in blue, include Willmore Wilderness area and Kananaskis Country. National protected areas, in green, include Banff, Kootenay, and Yoho National parks (the National Parks Complex) of Alberta, Canada.

We repurposed photographic data from three collaborative studies examining wolverine distribution in the Canadian Rocky Mountains, conducted in three landscapes: the Willmore Wilderness Area, Kananaskis Country, and Banff, Kootenay, and Yoho National Parks (National Parks Complex [NPC]; Fig. 1) (Fisher et al. 2011, 2013; Clevenger and Baruetto 2014; Fisher and Bradbury 2014; Heim 2015). All three areas exhibit rugged topography with mountains ranging above sea level from 825 m valley bottoms to 4000 m summits, and mid‐elevation conifer forests in between dominated by Engelmann spruce (*Picea englemannii*), Subalpine fur (*Abies lasiocarpa*), and Subalpine larch (*Laryx lyallii*). Diverse mammalian carnivore communities and prey communities inhabit all three landscapes (Fisher et al. 2011; Heim 2015).

The three areas vary marginally in terms of topography, persistent spring snow, and natural landcover (Fisher et al. 2013; Heim 2015); they differ strikingly in the degree of anthropogenic development. The Willmore Wilderness is fully protected from landscape development and has only horse and foot trails within it, with all terrain vehicle access limited to fur trappers. The NPC is a complex of nationally protected parks with concentrated, intensive development for tourism and transportation in the valley bottoms, little to no development throughout most of the landscape, and no trapping. Adjacent to NPC, Kananaskis Country is managed by various land‐use directives ranging from tourism and recreational activities to industrial development, such as petroleum extraction and forest harvesting, as well as historic and current fur trapping.

### Field protocols and data collection

We surveyed wolverines monthly at sites deployed in a systematic design comprised of 12 × 12 km^2^ grid cells imposed upon the study area. At each site, we surveyed wolverines with a combination of remote camera trapping (Burton et al. 2015) and noninvasive genetic tagging (NGT; Waits and Paetkau 2005) wherein the camera photographed the hair trap used for capturing genetic material, as well as the surrounding area. Wolverine occurrence was recorded using Reconyx^™^ digital cameras triggered by heat‐in‐motion (models RM30, PM30, PC900; Reconyx, Holmen, WI) at sites baited with a whole frozen beaver carcass, nailed to a tree wrapped in barbed wire, to capture hair. This double‐sampling approach allowed us to quantify error in NGT samples (Fisher and Bradbury 2014). Having a quantifiably low detection error (Fisher and Bradbury 2014), this technique facilitated a primary objective: estimating wolverine abundance and distribution (e.g., Fisher et al. 2013). However, the camera dataset also allowed us to observe the behaviors of wolverines in the vicinity of, and interacting with, the baited tree at each site.

We deployed cameras in December through March in the Willmore Wilderness (winters 2006/2007 and 2007/2008), where each site was deployed for one winter, and January through April in the NPC (2012, 2013) and Kananaskis Country (2011, 2012), where some sites were deployed for one winter and some for both. We used a dataset comprised of the first year of data from each of 164 sites across the three areas (Willmore = 66, NPC = 52, Kananaskis Country = 45), spanning 24,000 km^2^, with 100 uniquely identified individuals (Willmore = 28, NPC = 64, Kananaskis Country = 8) – the largest North American dataset on wolverine distribution extant (Fisher et al. 2013; Clevenger and Baruetto 2014; Heim 2015). We quantified behaviors from all monthly surveys at each site.

### Quantifying behaviors

Little is known about wild wolverine behavior (Banci 1994). This required that we posit four fundamental assumptions about wolverine behavior at these sites, based on established wildlife behavior theory and observations. First and most generally, we assumed that as scavenging carnivores ranging across vast areas, behavioral plasticity is a key component to wolverine life history and that individuals exhibit different behaviors under different conditions (e.g., Komers 1997; Sih et al. 2004a). Second, we assumed that climbing a tree to acquire the bait posed a perceived risk to wolverines. The nature of this risk is unknown; it may be due to neophobia of the trap itself, or perceived risk of being away from visual cover and escape cover, or some other unknown factor. Third, we assumed that time spent at the baited site posed a risk to wolverines. Several other (larger) carnivore species are attracted to bait (Long et al. 2007; Fisher et al. 2011) and an interspecific encounter can lead to wolverine mortality, as suggested by intraguild predation rates in Krebs et al. (2004). Fourth, we assumed that individual wolverine response to these risks is not static but instead varies in space as a result of behavioral plasticity Komers (1997), a natural corollary of optimization theory (Pyke et al. 1977; Krebs 1978).

Based on these assumptions, we quantified four metrics: (1) the probability that a wolverine detected at a site would climb the baited tree; (2) the latency (time in minutes) of a wolverine to show up at a site; (3) the latency (time in minutes) for a wolverine to climb the baited tree, given it climbed; and (4) the total time (in minutes) spent at a site. There may be other, more subtle behaviors such as “head‐lifting” risk‐related behaviors (e.g., Lima and Dill 1990; Lima and Bednekoff 2014) but we used these four because we contend their obviousness and ease of quantification make them conservative metrics less prone to subjectivity and observer error.

**Figure 2:**
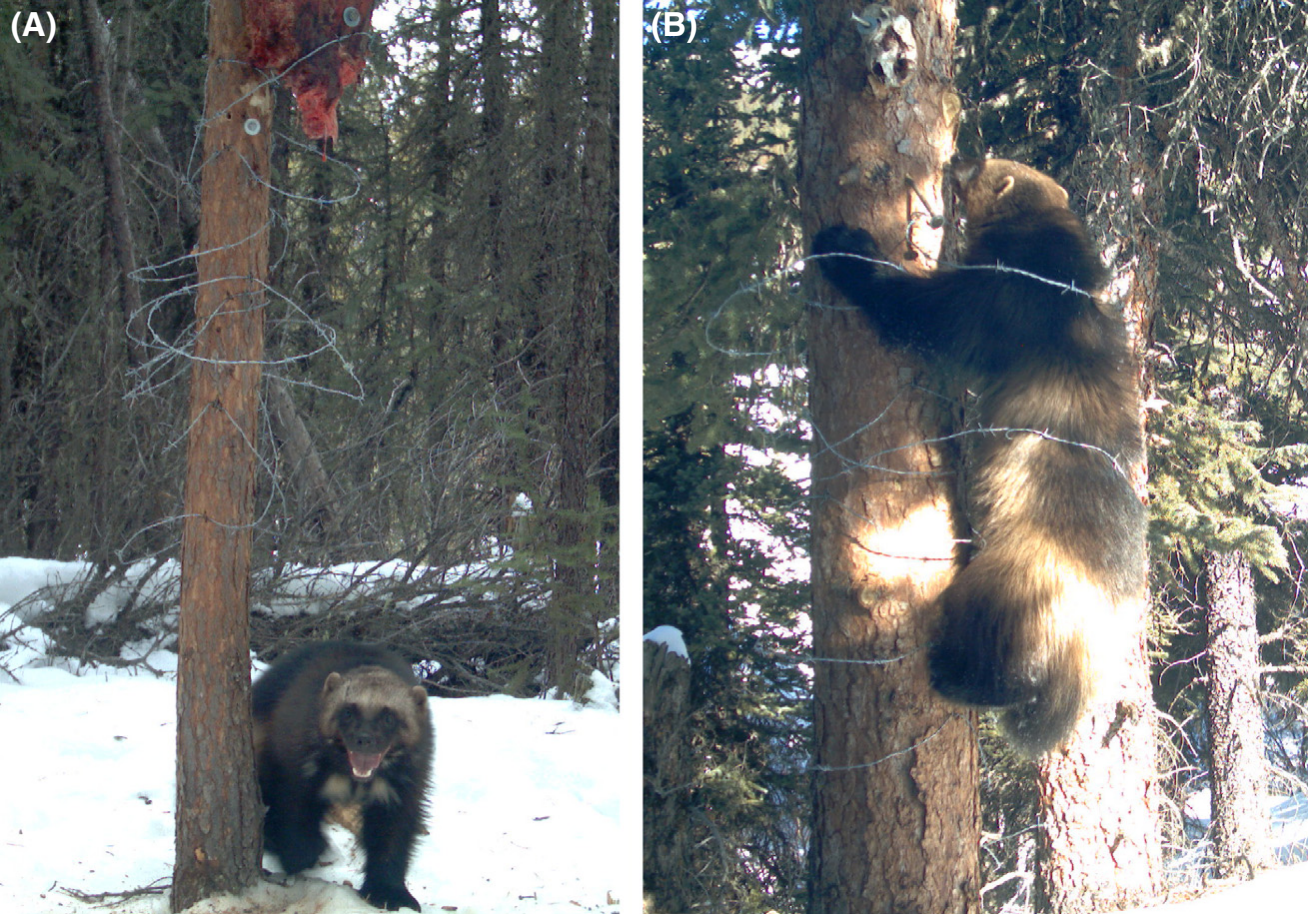
Although wolverines posses distinctive chest markings (A), wildlife cameras were not always positioned to identify individuals, individuals did not always face the camera (B), and photographed individuals did not always leave hair for genetic identification.

We used digital infrared remote camera images from each site to measure these four metrics. When triggered, cameras took five photographs at 1‐s intervals, repeated at each detected movement. Images therefore comprise a short time‐lapse video of wolverine behavior at each site and each visit. For each time series (month) of photographs, we used a standardized protocol to record the time elapsed between camera setup and wolverine detection (latency to detection; min), the time at which the wolverine climbed the tree (latency to climb; min), and the time spent at the site before moving off (total event duration; min). Although wolverines possess distinctive chest markings, our cameras were not always positioned to identify individuals (e.g., Magoun et al. 2011; Fig. 2), and photographed individuals did not always leave hair for genetic identification (Clevenger and Baruetto 2014; Fisher and Bradbury 2014; Heim 2015). Thus, our question does not address individual behavior; rather we ask whether wolverine behavior, averaged across the population, changes across a gradient of human footprint. As wolverines are social scavenging carnivores with vast territories (Krebs et al. 2007), we assumed that an individual would consume or cache the bait within a few hours and move on to another location. Therefore, we recorded a different behavioral “event” to occur at a site after a 6‐h period of site inactivity. At each event, we measured the three behavioral metrics, and averaged each metric among events, yielding a single value at each site. This conservative method makes it harder for us to find a signal (reducing Type I error) by decreasing the total variation in behavior.

### Habitat analysis

Digital map inventories from the Alberta Biodiversity Monitoring Institute (ABMI; Human Footprint Map 2012, and National and Alberta Provincial Parks’ geo‐databases) were accessed to quantify 16 anthropogenic landscape features: percent area of urban landcover, cultivation, disturbed vegetation, rural residential, petroleum extraction well sites, forest harvesting cut blocks, industrial sites, mine sites, pipelines, transmission lines, petroleum exploration seismic lines, roads, and rail lines. Based on extensive exploratory analyses (Heim 2015), we merged noncollinear features in ArcGIS 9.3.1 (Environmental systems research Institute, Redlands, CA) into one “cumulative human footprint” variable. We calculated the percent cover (% area) of human footprint at a 5000‐m buffer around each site (Fisher et al. 2013). We also calculated the linear feature density of seismic lines, pipelines, transmission lines, roads, and rail lines (km/km^2^) around each site, at this same extent. Although there is some redundancy in the measures, linear features are the most spatially extensive form of disturbance in this region, and Heim (2015) suggests wolverines respond to linear features more strongly than patch features.

### Statistical analysis

We tested for correlations between behavioral variables and found none (Table 1). We conducted two analyses to investigate the association between wolverine behavioral variation and human footprint. First, we calculated the proportion of sites where a wolverine was present and climbed the baited tree. We used a generalized linear model (binomial error distribution, logit‐link function) in R (R Foundation for Statistical Computing 2012) to estimate the probability of climbing in relation to cumulative human footprint (% area) and density of linear features (km/km^2^), as these variables test the hypotheses that wolverine behavior changes with increasing landscape modification. Second, we conducted two Levene's tests of equal variances (Levene 1960), using the “car” package in R (Fox and Weisber 2014), to test for behavioral variation, and variation in human footprint, among the three study areas.

**Table 1:** Correlations between recorded behavioral variables collected at each study site. Numbers below the diagonal represent Pearson's correlation coefficients, where no correlation was significant (*α *< 0.05). Numbers above the diagonal represent degrees of freedom

	Latency to detection	Latency to climb	Total event duration
Latency to detection		87	98
Latency to climb	−0.04		86
Total event duration	−0.03	0.05	

To weigh support for our hypothesis, we wished to model latency to climb the tree in relation to the several landscape variables we quantified, and rank these models in an information‐theoretic approach (Burnham and Anderson 2004). However, the distribution of these data did not lend themselves to any of the available generalized linear models and links (Crawley 2007), and the discrepancy in sample sizes between study areas did not lend the data to linear mixed effect models (Pinheiro et al. [Link]), generalized least squares regressions (sensu Zuur et al. 2009), or nonlinear least squares regressions (Bates and Chambers 1992). Upon inspecting the data, we instead posed the ad hoc hypothesis that these data were represented by two distributions in this dataset, representing two linear relationships between the response and predictor variables. In plain terms, we suspected that wolverines were behaving one way in response to one range of the anthropogenic footprint features, and differently to another range of these features. We tested this ad hoc hypothesis with a piecewise (or segmented) regression analysis, in the R package SiZer (Sonderegger 2015) to identify the point at which a linear model with cumulative human footprint as a predictor variable, and latency to climbing as the response variable, would have the smallest mean square error (Toms and Lesperance 2003; Sonderegger 2015). We then regressed two linear models: one using predictor data (average latency to climb) smaller than the derived break point and the other using predictor data (average latency to climb) larger than the break point, to demonstrate the change in wolverine behavior before and after this break point.

## Results

**Figure 3:**
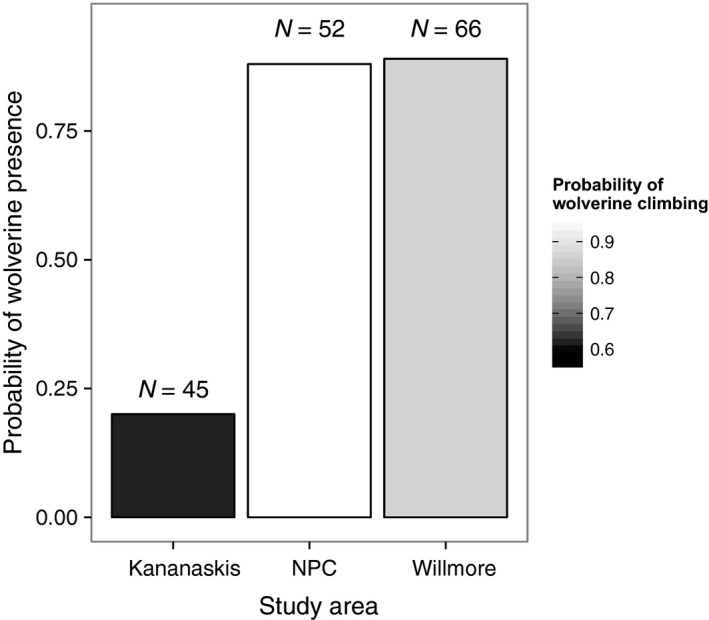
The probability of wolverine presence and climbing varied across three study areas in the Alberta Rocky Mountains, Canada. Wolverine had the lowest probability of occurrence and of climbing a baited tree in Kananaskis Country. Sample sizes indicate the number of sites sampled within each study area.

Cumulative human footprint varied among all three study areas (Levene's test: *F*_2,160_ = 51.82, *P* < 0.0001), with the greatest landscape modification found in the Kananaskis Country region compared with the other two areas (Kananaskis 8.09 ± 0.77% area; NPC 1.47 ± 0.50% area; Willmore 0.0005 ± 0.0001% area). Wolverine climbing also varied across the three study areas, with wolverines in Kananaskis Country, the least likely to climb a baited tree when detected at a site, at 56% (5/9 sites). In the Willmore Wilderness, 88% (46/52 sites) of the wolverines detected at a site climbed the bait tree and in the NPC, 96% (44/46 sites) of those detected climbed the bait tree (Fig. 3).

**Figure 4:**
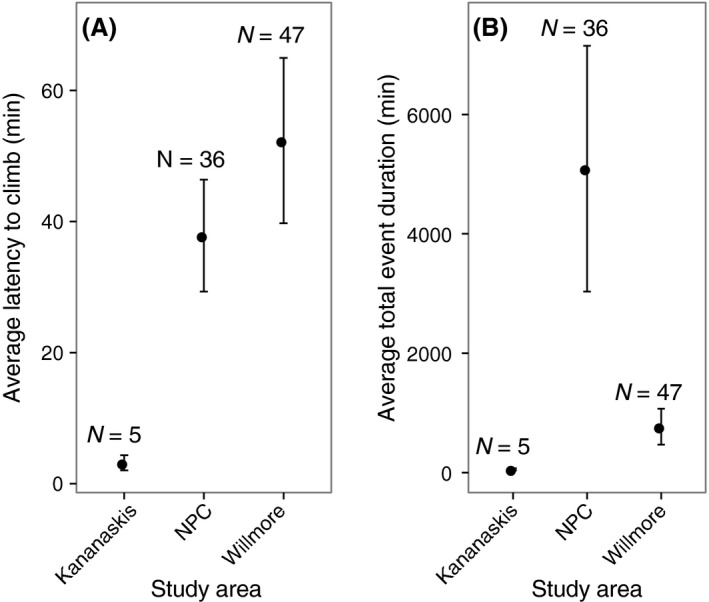
Both the absolute value (mean) and variation (SE) of the latency of a wolverine to climb a baited site (A) and the total duration of each event (B) were significantly different between landscapes with different degrees of disturbance. Sample sizes indicate the number of sites with wolverine present within each study area.

Behaviors varied among all three study areas (Fig. 4). The average latency of climbing varied (Levene's test; *F*_2,86_ = 3.69, *P* = 0.03); climbing was the fastest and least variable in Kananaskis, and slower and more variable in Willmore. The total event duration also varied between study areas (Levene's test; *F*_2,97_ = 13.35, *P* < 0.001); events were fast and least variable in Kananaskis. Total event duration was the longest and most variable in the NPC (Fig. 4). In the less human‐modified Willmore Wilderness and National Park Complex, wolverines displayed a greater range of behavior: there was on average 16.5 and 11.9% greater behavioral variation in their latency to climb, and 13.8 and 37.5% greater behavioral variation in the duration spent at a site (respectively) than in Kananaskis Country (Fig. 4).

**Figure 5:**
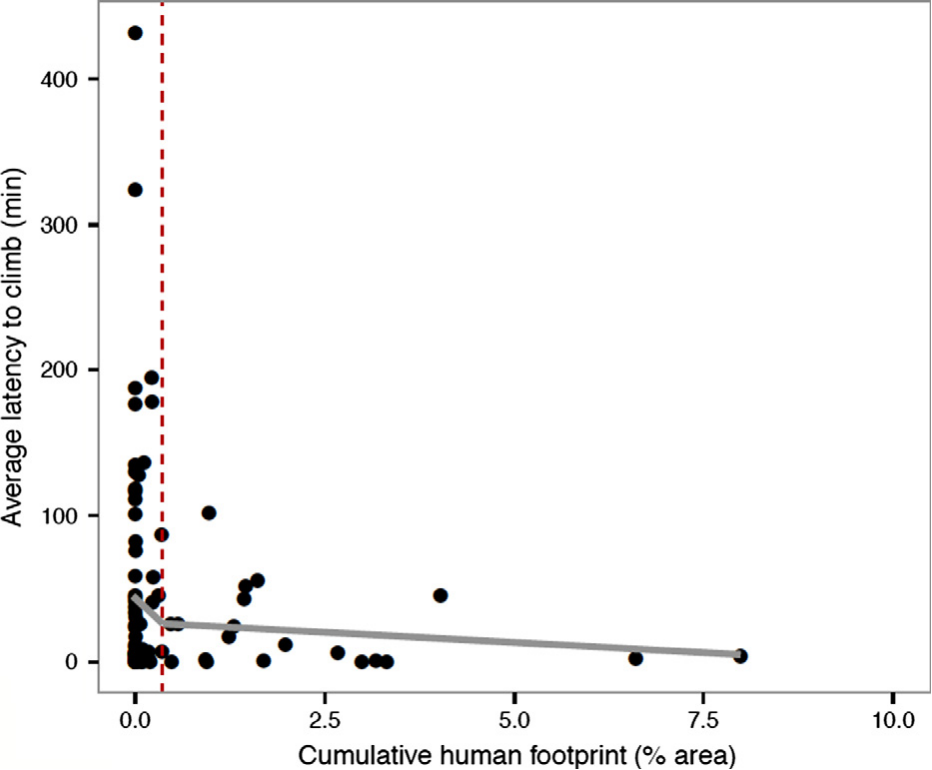
Wolverine decreased their average latency to climb across a range of human footprint values. An ecological threshold in behavior is represented by the red‐dashed line; when greater than 0.35% of area is covered by human footprint, wolverine significantly change their behavior, as represented by the different slopes of the gray lines before and after this threshold.

Wolverines’ climbing behavior changed at 0.35% human footprint, as suggested by the piecewise linear model regressing average latency to climb (min) as a function of cumulative human footprint (% area; Fig. 5). Average latency to climb decreased across the range of human footprint values, but this trend was significantly greater in areas with human footprint measuring less than 0.35%, represented by the linear model *y* = 49.87 − 50.46(*x*), than in areas measuring more than 0.35%, represented by the linear model *y* = 23.95 − 1.00(*x*) (Fig. 5).

There was no linear relationship between cumulative human footprint and the probability of climbing the baited tree (generalized linear model: *N* = 113, *Z* = −0.33, *P* = 0.74). Linear features correlate to human footprint (*r* = 0.37, df = 162, *P* < 0.001), but also do not predict the probability for a wolverine to climb the baited tree (generalized linear model: *N* = 113, *Z* = −1.33, *P* = 0.19).

## Discussion

Wolverines behaved differently in heavily human‐modified landscapes than in lightly human‐modified or protected landscapes. The time that wolverines spent at a site was less in Kananaskis Country compared with the other two areas. This difference is not subtle. In the less human‐impacted Willmore Wilderness and NPC, some wolverines lingered for hours at a site, and some wolverines went in and out quickly. However, in Kananaskis Country, wolverines rarely lingered. Instead, they consistently arrived at a site quickly, climbed quickly, and left quickly. We contend that this pattern, manifested over a very large landscape, a range of human footprint, and over 100 individuals, is consistent with evidence for spatial variability in behavior correlated with increasing human footprint.

### Hypothesized drivers of wolverine behavior

In developed areas, wolverines were constrained to a behavior where they climbed quickly (if they climbed at all), and left quickly. If our assumptions are correct – that climbing an exposed baited tree and lingering at a site represent a risk – then we provide good evidence that wolverines perceive human‐modified landscapes as riskier. What might wolverines be afraid of, if they are indeed afraid? Most LOF studies are based on predation risk as a direct mechanism (Laundré et al. 2010; Matassa and Trussell 2011). Some studies suggest the presence of humans can induce a similar response, increasing perceived mortality risk (Ciuti et al. 2012a). Neophobia is a common phenomenon among some taxa (Sih et al. 2004a,b; Real et al. 2007) and wolverines may experience this as well. Neophobia might explain the avoidance of roads, linear features, and other disturbed areas that are pervasive in wolverine literature (Rowland et al. 2003; Krebs et al. 2007; Fisher et al. 2013).

There are other contenders for mechanisms driving spatial patterns in wolverine behavior. In general, animal behavior can vary with fluctuations in population density (Sih 1984; Dantzer et al. 2012). Across these three study areas, variation in behavior differs, but population density is similar between two of the study areas: the Willmore Wilderness and the NPC (Fisher et al. 2013; Clevenger and Baruetto 2014). In contrast, wolverine occurrence (a surrogate for density) is much lower in Kananaskis Country (Heim 2015). If density were the sole driver, we may not have detected differences between the Willmore Wilderness and NPC. Because density changes with degree of human footprint, teasing these two factors apart may prove difficult.

Behavior can also vary with the degree of intraguild competition (Amarasekare 2003). Heim (2015) showed that wolverines were less likely to occur at sites with an increasing probability of coyote (*Canis latrans*) and red fox (*Vulpes vulpes*) occurrence and hypothesized that competition may be a factor influencing the differential response to human footprint between wolverine and mesocanids. The presence of these potentially competing species varies with human footprint, requiring more research to tease apart these mechanisms. Trapping and food availability (Pyke et al. 1977) may also be drivers of behavioral variation. Trapping can induce significant mortality (Krebs et al. 2004), and there may be selection for animals that can avoid traps. However, wolverines are trapped in both the Willmore Wilderness and Kananaskis Country, but wolverine detected in the Willmore Wilderness had a greater range of behavior than did wolverine detected in the Kananaskis Country region. Based on an assessment of camera data on prey communities, forage availability does not significantly differ between these three study areas.

### Caveats

Variation in wolverine behavior could be caused by several sources, but we are able to rule out some competing hypotheses. First, the signal we recorded is not likely due to detection error. Given that the probability of wolverine detection (sensu MacKenzie 2006) via cameras approached 1.0 in the Willmore Wilderness (Fisher and Bradbury 2014), and was similar in NPC and Kananaskis Country (Clevenger and Baruetto 2014; Heim 2015), we are confident that the majority of wolverine individuals were sampled using our field methods. Second, despite relatively equal sampling efforts in all three areas, there were fewer wolverines occurring in Kananaskis Country than in the Willmore or NPC, resulting in fewer behavioral observations there. If these observations came from a nonrepresentative sample of KC wolverines more risk‐averse than others, a false signal would be generated. However, we have no reason to suspect that is the case due to very high detectability of wolverine using our methods (Fisher and Bradbury 2014). Third, we recognize that our assumptions about what constitute risk‐averse behavior for wolverines may be completely incorrect. If these assumptions were not upheld, we would not expect to detect a behavioral signal that varies in space. However, we did find a signal, thus providing evidence to support our assumptions, which were founded in, and consistent with, behavioral theory. Wolverine behavior in the wild has never been assessed before, and spatial variation in behavior is a newly emerging subdiscipline (Lima and Zollner 1996). Moreover, proving risk perception can sometimes be contentious, despite a great body of work on the subject (Lima and Dill 1990). It might instead be the case that wolverines are quicker to climb, and quicker to leave, when conditions are good, although we could not formulate a reasonable guess why this might be. Given this is the first examination of wolverine behavior in the wild, we contend that the spatial pattern is worth considering, and the assumptions worth testing.

In summary, we have shown that there is spatial variability in wolverine behavior, and that this variability corresponds to increases in human footprint. Potential sublethal effects, if any, that such a landscape may impact upon wolverines have yet to be investigated. More importantly however, we contend that the conservation implications of this spatial variability may shed some light on the contentious debate about the mechanisms driving wolverine decline.

### Ecological and conservation implications

Human‐driven landscape‐scale changes have been widespread (Vitousek et al. 1997) and associated with rapid mammalian population decline and range contractions across North America for the past century (Woodroffe 2000; Laliberte and Ripple 2004), with no anticipation of slowing (Woodroffe 2000; Carroll et al. 2004; Cumming 2007). However, for many species the ultimate causes of range contractions are still unclear. Despite a rapidly emerging body of research, the mechanisms of wolverine range contractions remain contentiously in debate, to the detriment of conservation. Wolverines have twice been denied protection under the Endangered Species Act by the United States Fish and Wildlife Service, due (in part) to this debate. Wolverines’ association with persistent spring snow (Copeland et al. 2010; Clevenger and Baruetto 2014) has led some to contend that decreasing spring snow pack resulting from climate change primarily limits wolverine populations and distributions (Brodie and Post 2009; McKelvey et al. 2011; Inman et al. 2012). Although wolverine occurrence varies to some degree with snow pack across our study landscapes, it varies more strongly with linear features (Fisher et al. 2013; Heim 2015), mirroring past research showing negative responses to anthropogenic disturbance (Krebs et al. 2007). Before now this debate has compared spatial patterns of occurrence, which can result from multiple ecological processes. Behavior provides a different measure of response to disturbance than does occurrence. The concordance between behavioral response to linear features and large‐scale distribution lends support to human footprint as a driver of habitat suitability. If wolverines were driven only by snowpack we would expect no behavioral signal.

Wolverines’ behavioral shift in association with human footprint is additional evidence implicating landscape development as one of several mechanisms of population decline and range contraction. We echo others in advocating that conservation research should adopt several analytical approaches – population analysis, distribution analysis, behavioral analysis, etc. – to disentangle mechanisms of decline in complex landscapes.

## Conflict of Interest

None declared.
